# Reducing post-tonsillectomy haemorrhage rates through a quality improvement project using a Swedish National quality register: a case study

**DOI:** 10.1007/s00405-018-4942-3

**Published:** 2018-03-24

**Authors:** Erik Odhagen, Ola Sunnergren, Anne-Charlotte Hessén Söderman, Johan Thor, Joacim Stalfors

**Affiliations:** 1 Department of Otorhinolaryngology, Södra Älvsborgs Hospital, Brämhultsvägen 53, 501 82 Borås, Sweden; 2 Institute of Clinical Sciences, Sahlgrenska Academy at the University of Gothenburg, Gothenburg, Sweden; 3 Department of Otorhinolaryngology, Ryhov County Hospital Jönköping, Jönköping, Sweden; 4 Division of Clinical Science, Intervention and Technology, Karolinska Institutet, Stockholm, Sweden; 5 The Jönköping Academy for Improvement of Health and Welfare, Jönköping University, Jönköping, Sweden; 6 Sheikh Khalifa Medical City, Ajman, United Arab Emirates

**Keywords:** Quality improvement project, Post-tonsillectomy haemorrhage, Tonsillectomy, Healthcare quality improvement

## Abstract

**Purpose:**

Tonsillectomy (TE) is one of the most frequently performed ENT surgical procedures. Post-tonsillectomy haemorrhage (PTH) is a potentially life-threatening complication of TE. The National Tonsil Surgery Register in Sweden (NTSRS) has revealed wide variations in PTH rates among Swedish ENT centres. In 2013, the steering committee of the NTSRS, therefore, initiated a quality improvement project (QIP) to decrease the PTH incidence. The aim of the present study was to describe and evaluate the multicentre QIP initiated to decrease PTH rates.

**Methods:**

Six ENT centres, all with PTH rates above the Swedish average, participated in the 7-month quality improvement project. Each centre developed improvement plans describing the intended changes in clinical practice. The project’s primary outcome variable was the PTH rate. Process indicators, such as surgical technique, were also documented. Data from the QIP centres were compared with a control group of 15 surgical centres in Sweden with similarly high PTH rates. Data from both groups for the 12 months prior to the start of the QIP were compared with data for the 12 months after the QIP.

**Results:**

The QIP centres reduced the PTH rate from 12.7 to 7.1% from pre-QIP to follow-up; in the control group, the PTH rate remained unchanged. The QIP centres also exhibited positive changes in related key process indicators, i.e., increasing the use of cold techniques for dissection and haemostasis.

**Conclusions:**

The rates of PTH can be reduced with a QIP. A national quality register can be used not only to identify areas for improvement but also to evaluate the impact of subsequent improvement efforts and thereby guide professional development and enhance patient outcomes.

## Introduction

Tonsillectomy (TE) is one of the most frequently performed ENT surgical procedures, with over 700,000 operations performed in the United States each year [1]. In Sweden, with almost 10 million inhabitants, approximately 13,500 tonsil procedures are performed every year, half of which are TEs [2]. There are two main indications for tonsil surgery: (1) upper airway obstruction in children resulting in sleep-disordered breathing, and (2) infection-related problems (recurrent tonsillitis, chronic tonsillitis or peritonsillar abscess) [3]. Patients undergoing tonsil surgery due to upper airway obstruction are typically younger (incidence peaks at ages 3–5 years) and predominantly male; in contrast, patients undergoing tonsil surgery because of infection-related problems are typically older (incidence peaks at 16–18 years) and predominantly female [4].

Post-tonsillectomy haemorrhage (PTH) is the most feared complication of TE. A PTH is a potentially life-threatening event that often requires acute re-admission to hospital and sometimes a return to theatre. Reported rates of PTH vary in the literature; recent large studies indicate a range between 6 and 15% [5–8]. Fatal outcomes after PTH are rare, but should not be overlooked: a large Swedish cohort study documented a mortality rate after tonsil surgery (including both total and partial TE) of 1/40,000 [9]. In Austria, five children below the age of 6 years died after severe PTH in 2006–2007 [10]. Thus, the rate of PTH is of one the most important quality and safety indicators in tonsil surgery.

The National Tonsil Surgery Register in Sweden (NTSRS) was initiated in 1997 by The Swedish Association for Otorhinolaryngology, Head and Neck Surgery. The aim of the NTSRS is to monitor patient-related outcomes (e.g., symptom relief after surgery), complications, and clinical practice patterns to identify trends, initiate and perform research projects and stimulate local clinical improvement programmes. The NTSRS collects data on demographics, level of care (inpatient/outpatient), indication for surgery, dissection technique, haemostasis technique, incidence of postoperative haemorrhage and patient-reported outcome measures regarding postoperative pain, infections, haemorrhage, and symptom relief. The data management procedures of and results from the NTSRS have been described previously [3, 11–13].

The NTSRS is managed by a steering committee of experts in the field of tonsil surgery. In 2013, the NTSRS covered 81.2% of all patients who underwent tonsil surgery for benign indications [2]. Since the start of the register, all participating ENT centres have had complete access to their own data, enabling in-depth analyses that include comparisons of processes and outcomes to the Swedish average rates. A public annual report has been published since 2012 containing analyses and comparative data from every participating ENT centre. The annual reports have revealed wide variations in PTH rates among ENT centres, with a range from 0 to 25% [14]. The NTSRS has also shown that the same centres have placed at the top or bottom of the PTH rate list over several consecutive years. These persistent differences in PTH rates among surgical centres in Sweden indicate a potential gap between local practice and best practice and show that centres with high rates of PTH have the potential to reduce these rates.

The reduction of PTH rates in centres with high rates was identified by the NTSRS steering committee as a high-priority goal for a structured quality improvement project (QIP). Examples from other clinical fields have shown that structured QIPs using a national quality register can improve clinical results [15–17].

Therefore, in 2013, the NTSRS initiated a QIP to decrease the incidence of PTH. The project was planned by the NTSRS steering committee and managed by two of the authors (AHS, JS) with support from quality improvement experts from the Centre of Quality Registers Västra Götaland in Sweden. The project received financial support from the National Programme for Quality Registries.

The aim of the present study was to describe and evaluate the multicentre QIP initiated to decrease PTH rates.

## Materials and methods

### Settings and design of the QIP

In 2013, six surgical centres, all with PTH rates above the Swedish average rate, were invited and agreed to participate in the QIP. The participating centres were all public county hospitals with ENT residency programmes. At each centre, the head of the department appointed an ENT surgeon as a local project manager. All project managers were given 2 weeks free from their regular work to participate in the project. The project started in October 2013 and ended in April 2014.

The QIP started with a 2-day workshop at which the local project managers were updated on best practices and evidence-based medicine regarding tonsil surgery. During the workshop, the participants mapped the tonsil surgery process at their respective centres. Then, each participant created an individual action plan based on the discrepancy between best practices and local practices and containing remedial actions to reduce PTH.

The workshop included the following:

- Lectures on quality improvement tools, such as the plan-do-study-act method and the Ishikawa (cause-and-effect) diagram [18].
- Presentations of clinical practice in different countries/centres and the related outcomes.
- Update on scientific evidence and best practice regarding tonsil surgery and PTH. The benefits of cold instruments for both dissection of the tonsils and haemostasis during surgery was propagated based on the substantial, but under-applied scientific evidence that cold techniques for both dissection and haemostasis reduce PTH rates [11, 19, 20].
- Training on how to analyse and use NTSRS datasets to characterize local clinical practice.
- Planning clinical improvement efforts for each centre based on the gap between current local clinical practice and best practice.

Back at their respective centres, the local project managers presented the improvement plans to the heads of their departments and their fellow ENT surgeons. Local improvement plans were agreed upon and implemented as an integrated part of the department’s regular work. The improvement plans often included other staff members, such as theatre nurses and anaesthetic personnel.

The project lasted 7 months. The timing and content of the implementation process differed among the participating surgical centres, and not all changes were implemented at the same time. During the project period, the NTSRS project leaders regularly supported the local project manager by phone and e-mail. At a follow-up meeting after 7 months, each centre reported the changes they had made in practice. The NTSRS was reviewed to assess whether these changes had led to subsequent changes in outcomes, such as decreased PTH rates. The official project ended with this meeting, but the efforts continued at the centres, and both local stakeholders and the NTSRS steering group could continue to monitor the results online via the NTSRS.

### Study design and data sources

A case study design was used to describe and evaluate the QIP since such designs lend themselves well to illuminating multifaceted changes over time in relation to different contexts [21].

To evaluate the impact of the 7-month QIP, the authors identified a control group consisting of 15 surgical centres in Sweden that had PTH rates similar to those of the 6 QIP centres (8–17%) 12 months prior the start of the programme. Data for the 12 months prior the start of the QIP (baseline) were compared with the 12 months after the QIP (follow-up) for both groups.

The demographics of the study population and indicators for tonsil surgery were retrieved from the NTSRS. The NTSRS uses four questionnaires for collecting data (administered preoperatively, postoperatively, 30 days after surgery and 6 months after surgery), as detailed previously [3, 11, 13]. The outcome data for this study (PTH rates) were collected via a questionnaire completed by the patient 30 days after surgery. The response rate for the 30-day postoperative questionnaires was 53% in 2013. A more complete data set was desirable for PTH; therefore, data from the NTSRS was supplemented with data from the National Patient Register (NPR). The NPR is managed and administered by The National Board of Health and Welfare, a government agency under the Ministry of Health and Social Affairs. Registration in the NPR is mandatory by law for public and private care providers (except primary care) in Sweden. The NPR contains individually based information, including surgery and postoperative complications such as PTH [22]. The two registries were merged on an individual level using personal identity numbers to detect any PTH within 30 days after surgery. The methodology of merging data for PTH has been used for several years in the annual reporting of outcomes from the NTSRS [2, 14]. The merging of data was performed in collaboration with representatives from The National Board of Health and Welfare to ensure the integrity and validity of the data.

All the participating centres had written improvement plans describing the changes they intended to make in clinical practice. The plans were reviewed and analysed for this study to characterize and describe the types of improvement activities.

### Process indicators

The process indicators retrieved from the NTSRS included the techniques used for dissection and haemostasis. These techniques were classified into groups, “cold” and “hot”, based on whether the chosen surgical instruments added heat to the surgical field. Cold steel dissection was categorized as “cold dissection”, whereas coblation, diathermy scissors, ultracision and bipolar diathermy were categorized as “hot dissection”. “Cold haemostasis” was defined by the use of packs, ties and adrenaline infiltration, and “hot haemostasis” was defined by the use of bi- or monopolar diathermy. If any “hot” technique was used for dissection, the haemostasis technique was also considered “hot” [11].

### Outcome variable

The outcome variable for the project was PTH, which was defined in this study as bleeding from the throat that occurred after discharge and within 30 days from surgery and resulted in re-admission to hospital.

### Statistical analyses

The distributions of variables are given as numbers and percentages for categorical variables and as the mean, standard deviation (SD), median, minimum, and maximum for continuous variables. For comparisons between groups, we used Fisher´s exact test (lowest 1-sided *p* value multiplied by 2) for dichotomous variables, the Mantel–Haenszel chi-square test for ordered categorical variables, the chi-square test for non-ordered categorical variables, and the Mann–Whitney *U* test for continuous variables. For comparisons between groups, generalized estimating models were used to analyse PTH rates. *p* values for comparisons between groups for each time point and between time points within each group based on these analyses are shown for the variable “readmission for haemorrhage”. All significance tests were conducted at the 5% significance level. SAS Software Version 9 (SAS Institute, Cary, NC, USA) was used for all statistical analyses.

### Ethical considerations

The study was approved by The Regional Ethical Review Board in Gothenburg, Sweden (Reg. No. 257-14). Data management was handled according to Swedish law and regulations.

## Results

### Improvement activities

Six surgical centres (“Intervention group”) participated in the QIP. The improvement plans were unique for each surgical centre, although many features were the same across centres. Five main change themes emerged (Table 1). All the centres reported that they intended to change their surgical practice by minimizing the use of hot techniques. This included decreased use of bipolar diathermy for haemostasis (all centres) and the use of lower power settings for the bipolar diathermy device (five of the six centres). One centre that used coblation (a hot technique) prior to the QIP changed to cold dissection during the intervention period. Five centres reported that they would revise their strategy for pharmacological pain treatment. Four of the six centres aimed to improve their adherence to the national guidelines for pain treatment in paediatric patients. Five centres improved and updated their patient education by referring patients and caregivers to the website “tonsilloperation.se” for pre- and postoperative information. Published by the committee of experts that manages the NTSRS, this website contains practical information (in Swedish and other languages) about tonsil surgery for patients and caregivers. All the centres aimed to upgrade the surgical status of tonsillectomy (in Sweden, tonsil surgery is one of the first surgical procedures taught to residents and is often regarded as “a simple and common procedure”). Actions to elevate the status of tonsillectomy included improved education in tonsillectomy surgical technique for junior doctors and having discussions and experience exchanges about tonsil surgery in staff meetings.

**Table 1:** Themes of improvement activities and their use by the surgical centres

Themes of improvement activities	Borås	Falun	Karlstad	Norrbotten	Skövde	Västerås
**Change in surgical technique**						
Increase the use of cold dissection and haemostasis	X		X	X	X	X
Decrease the use of coblation and/or bipolar diathermy and/or bipolar scissors	X	X	X	X	X	X
Reduce the power settings for bipolar diathermy	X	X		X	X	X
**Revise the strategy for pharmacological pain treatment**						
Improve adherence to the national guidelines for pain treatment in paediatric patients		X	X	*	X	X
Revise the use of NSAIDs preoperatively		X		X		X
**Information for patients and caregivers**						
Improve postoperative information to patients		X	X	X	X	X
Introduce telephone follow-up			X	*	X	*
**Elevate the status of tonsillectomy**						
Improve training in the surgical technique of tonsillectomy among junior doctors	X	X	X	X		X
Discuss the quality improvement project in staff meetings	X	X	X	X	X	X

* Implemented before the quality improvement project

### Surgical and patient demographic characteristics

12 months before the QIP started (“baseline”; October 2012 to September 2013), the number of tonsillectomies performed at the surgical centres in the intervention group varied between 155 and 233; 1220 surgeries were performed. In the control group, a range of 17–372 surgeries was performed at each centre, with 1318 surgeries performed during the same period. Demographics and baseline patient characteristics are shown in Table 2. There were no gender or age differences between the groups; female patients were more common in both the intervention and control groups. There was a small but statistically significant (*p* = 0.0025) difference in the indication for surgery, with slightly more patients treated for infection-related problems in the control group at baseline. Outpatient TE was more common in the control group than the intervention group both at baseline and at follow-up.

**Table 2:** General characteristics, process indicators and outcome at baseline and follow-up

Variables	Group of surgical centres						Control versus intervention	
	Control group			Intervention group			Baseline	Follow-up
	Baseline	Follow-up	*p* value	Baseline	Follow-up	*p* value	*p* value	*p* value
Tonsillectomy cases	*n* = 1318	*n* = 1387		*n* = 1220	*n* = 942			
Surgical centres (*n*)	15	15		6	6			
Gender								
Male	572 (43.4%)	564 (40.7%)		517 (42.4%)	430 (45.6%)			
Female	746 (56.6%)	823 (59.3%)	0.16	703 (57.6%)	512 (54.4%)	0.14	0.63	0.019
Age (years)	19.8 (12.5) 18.5 (1.9; 76.9) *n* = 1318	20.5 (12.3) 19.1 (1.6; 72.6) *n* = 1387	0.082	20.2 (13.8) 18.1 (1.7; 78.0) *n* = 1220	21.3 (14.0) 18.8 (1.8; 77.8) *n* = 942	0.042	0.71	0.85
Indication								
Obstruction	379 (28.8%)	354 (25.5%)		370 (30.3%)	275 (29.2%)			
Infection	905 (68.7%)	993 (71.6%)		789 (64.7%)	609 (64.6%)			
Other indications	34 (2.6%)	40 (2.9%)	0.16	61 (5.0%)	58 (6.2%)	0.47	0.0025	< 0.0001
Level of care								
Outpatient	736 (55.8%)	919 (67.3%)		565 (46.3%)	450 (48.0%)			
Inpatient	582 (44.2%)	446 (32.7%)	< 0.0001	655 (53.7%)	487 (52.0%)	0.43	< 0.0001	< 0.0001
Missing value		*22*			*5*			
Process indicators								
Techniques for dissection/haemostasis								
Cold/cold	89 (6.8%)	70 (5.2%)		76 (6.2%)	138 (17.6%)			
Cold/hot	508 (38.7%)	597 (44.2%)		692 (56.7%)	601 (76.5%)			
Hot/hot	715 (54.5%)	685 (50.7%)	0.34	452 (37.0%)	47 (6.0%)	< 0.0001	< 0.0001	< 0.0001
Missing value	*6*	*35*			*156*			
Techniques for dissection								
Cold dissection technique	603 (45.8%)	673 (49.6%)		768 (63.0%)	743 (94.1%)			
Hot dissection technique	715 (54.2%)	685 (50.4%)	0.053	452 (37.0%)	47 (5.9%)	< 0.0001	< 0.0001	< 0.0001
Missing value		*29*			*152*			
Techniques for haemostasis								
Cold haemostasis technique	89 (6.8%)	82 (6.0%)		76 (6.2%)	146 (15.6%)			
Hot haemostasis technique	1223 (93.2%)	1295 (94.0%)	0.42	1144 (93.8%)	788 (84.4%)	< 0.0001	0.63	< 0.0001
Missing value	*6*	*10*			*8*			
Outcome								
Post-tonsillectomy haemorrhage, readmission within 1–30 days								
Readmission for haemorrhage, no	1183 (89.8%)	1236 (89.1%)		1065 (87.3%)	875 (92.9%)			
Readmission for haemorrhage, yes	135 (10.2%)	151 (10.9%)	0.59	155 (12.7%)	67 (7.1%)	< 0.0001	0.052	0.0025

For categorical variables, *n* (%) is presented. For continuous variables, mean (SD)/median (min; max)/*n*, is presented

### Process indicators

**Fig. 1:**
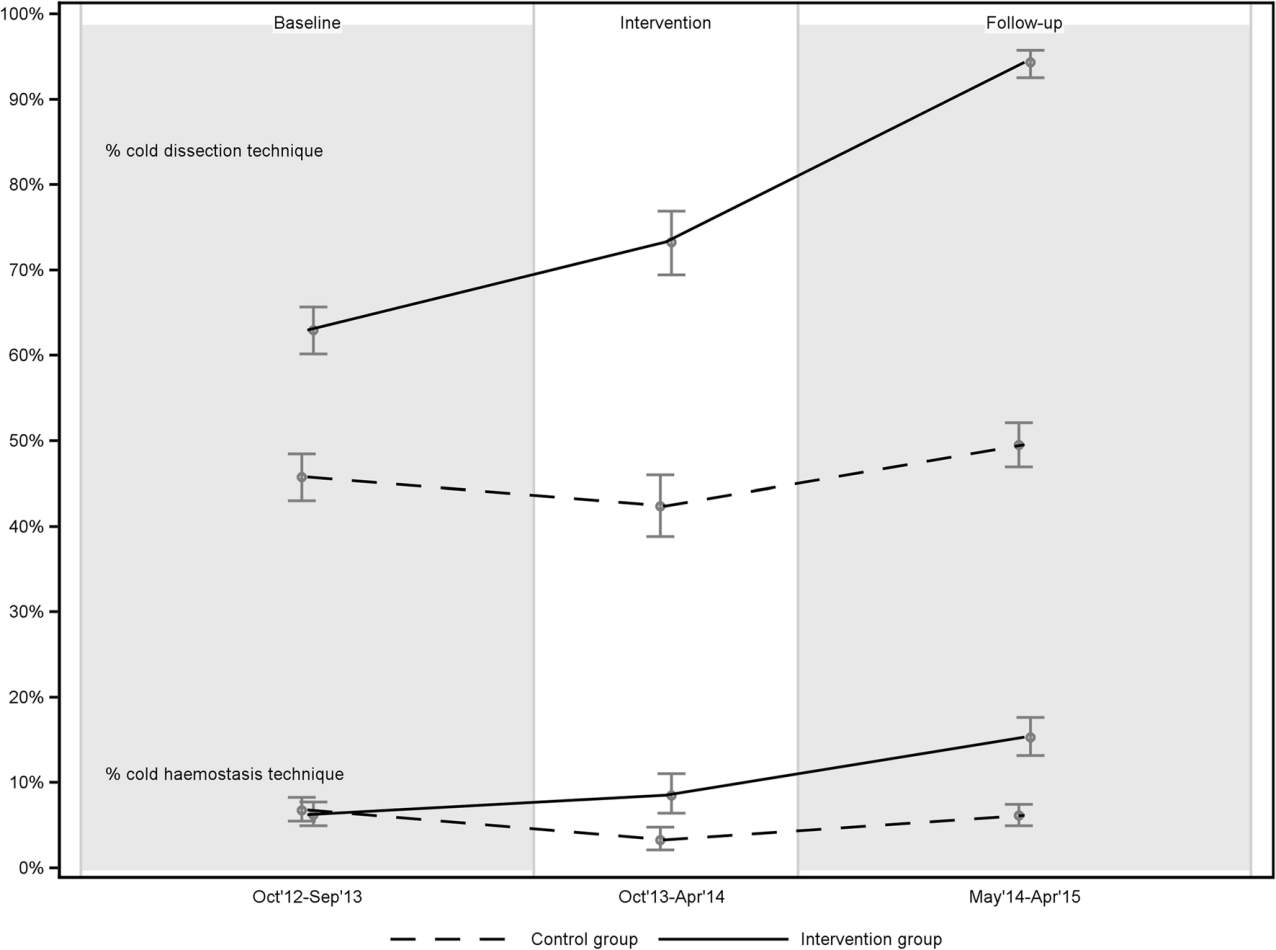
Techniques for dissection and haemostasis at baseline, intervention period and follow-up, displayed with 95% confidence interval

At baseline, the use of cold dissection techniques was more common in the intervention group (63.0% of all TEs) than in the control group (45.8%). There were no differences in haemostasis techniques. In the intervention group, there was a significant increase from baseline (63.0%) to the follow-up period (94.1%) in the use of cold dissection techniques. There was also an increase in the use of cold haemostasis techniques in the intervention group, from 6.2% at baseline to 15.6% at follow-up. The control group showed no significant changes in techniques for dissection or haemostasis from baseline to follow-up (Table 2; Fig. 1).

### Outcome

**Fig. 2:**
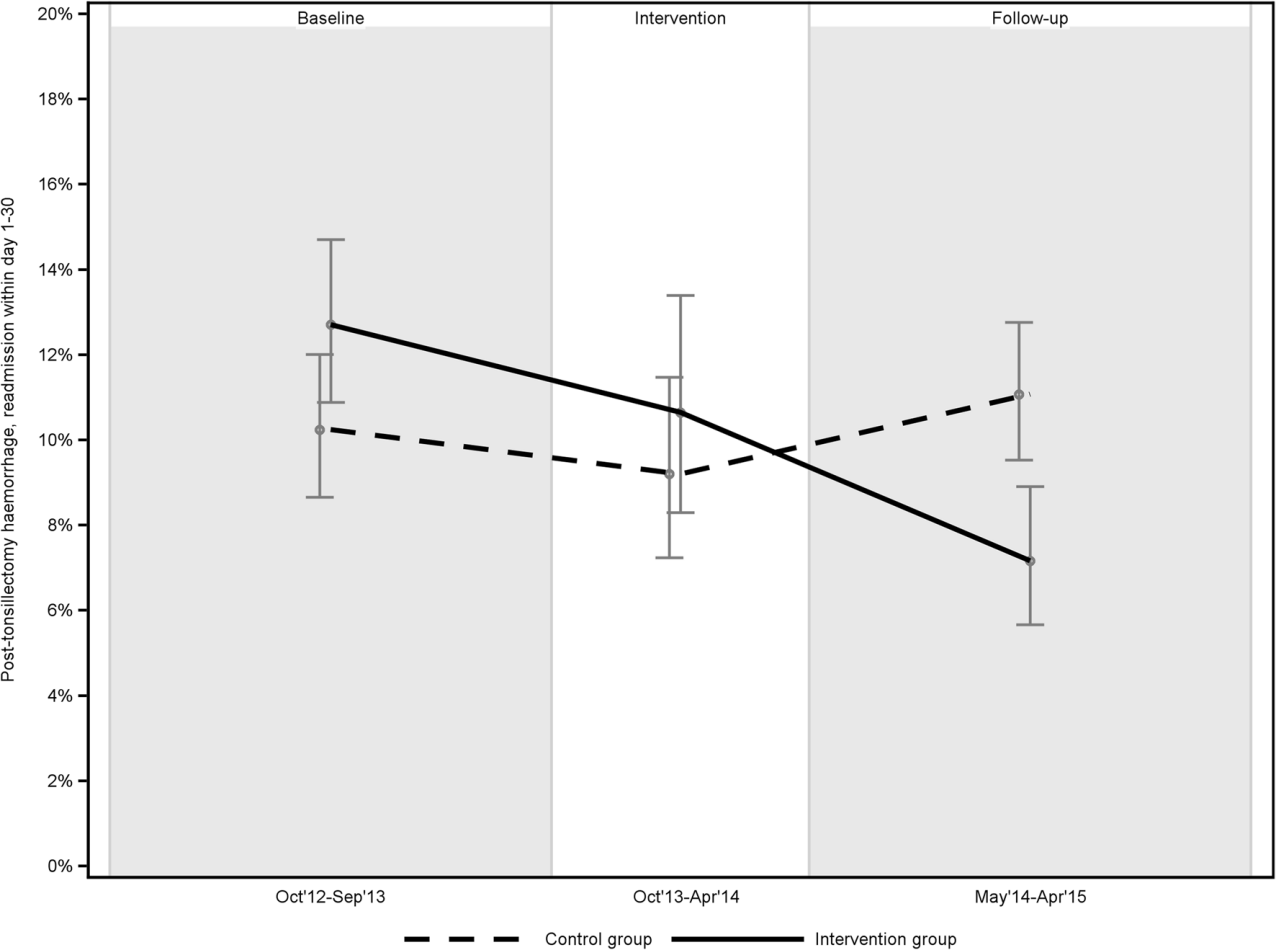
PTH rates at baseline, intervention period and follow-up, displayed with 95% confidence interval

There was no statistically significant difference between groups regarding the outcome variable, PTH rate, at baseline. In the intervention group, baseline to follow-up comparisons demonstrated a significant reduction of PTH rates, from 12.7 to 7.1%. The control group showed no change in PTH from baseline to follow-up. At follow-up, there was a statistically significant (*p* = 0.0025) difference between the intervention group (7.1%) and the control group (10.9%) regarding PTH (Table 2; Fig. 2).

## Discussion

Tonsillectomy is a common surgical procedure with well-established positive effects on several medical conditions. Tonsillectomy, like all surgical procedures, carries a risk of complications. The most important of these, postoperative haemorrhage (PTH), not only carries the risk of a fatal outcome, but is often a traumatic and negative experience for the patient and family. Furthermore, it places an avoidable burden on the health care system.

There are numerous publications on PTH rates that show wide variation in these rates, which indicates that many instances of PTH could be avoided [5–8]. This should inspire many ENT surgeons to review their own practices. This article demonstrates that it is possible to decrease PTH rates through a QIP – the intervention centres reduced the PTH rate from 12.7 to 7.1%, i.e., an average 5.6 fewer instances of PTH per 100 TEs. To the best of our knowledge, this QIP evaluation is the first of its kind. The NTSRS leadership concludes that QIPs can and should be used to decrease PTH rates, especially in surgical centres that persistently have higher rates of PTH than their peers. In Sweden, the NTSRS provides important support for initiating and evaluating such projects.

Drawing on the best available scientific evidence, cold techniques were promoted in this project. The QIP led to positive changes in the key process indicators: techniques for dissection and haemostasis. Participating local managers reported planning for such changes; subsequently, a significant increase in the use of cold dissection and haemostasis was documented in the register in the intervention group, but not in the control group. Having found no other plausible explanations for the clinically and statistically significant reduction in PTH rates (from 12.7 to 7.1%) among the QIP centres, we conclude that the QIP led to the reduction.

There are likely multiple reasons for this observed decrease in PTH rates. The reasons may also differ among the participating centres. It is not possible to describe or study all the factors contributing to this result due to the study design and the limitations of data collected in the NTSRS. Our results strongly indicate that the main contributors followed from the increased use of cold techniques for surgical dissection and haemostasis. However, other factors may have contributed in a web of influences, including the increased awareness of PTH among the clinical staff, the “upgrade” of the status of tonsil surgery and the improved surgical training. Furthermore, the engagement of local project leaders helped, as did the support from department heads and the tailoring of improvement plans to each centre’s local context, a key consideration in health care improvement [23, 24]. The possibility of releasing the local project manager from clinical work for 2 weeks was appreciated, and this time was used for analysis, lectures and implementing the improvement plan. For example, for an ENT department to change its surgical technique practices, it was necessary to review and sometimes change instruments and to educate the nurses and surgeons involved in the tonsil surgery process.

There is growing evidence that PTH and other complications, such as pain, are more common when hot techniques are used [25, 26]. At least three population-based studies have demonstrated that cold dissection and cold haemostasis result in lower rates of PTH, but hot instruments continue to dominate in clinical practice [5, 11, 19]. A British audit presented clear recommendations of the cold technique, but a follow-up study showed a relapse in the use of hot instruments [27]. In Sweden, the PTH rate has been unchanged on national level for the last 5 years, regardless of fact that the results from the NTSRS, including recommendations regarding the use of cold techniques, are published and distributed to all Swedish ENT surgeons annually [14]. However, the present study shows that a QIP can change entrenched habits.

### Methodological considerations

There are important limitations to this study’s generalizability. First, the six surgical centres that participated in the QIP all volunteered to participate, whereas the control group consisted of surgical centres with similar PTH rates that had not been invited to participate in the QIP. Volunteering equals self-selection and a potentially greater commitment to change than non-volunteers have, which might have impacted the result. However, the baseline measurements showed no or small significant differences in PTH rates and process indicators (dissection and haemostasis techniques), which indicates that the even if the volunteering surgical centres were more eager to improve, their eagerness had not reduced their PTH rates to near the national average before the start of the QIP. We believe that the QIP is the main explanation for the changes in surgical techniques and decreases in PTH rates observed in the intervention group. Furthermore, the will to improve is an essential precondition for improvement [28].

Second, although the control group consisted of centres with similar PTH rates, the two groups differed slightly in other baseline characteristics: The control group included surgical centres that performed relatively fewer TEs each year. The NTSRS data indicate, however, that there is no association between a centre’s volume of surgeries and PTH rates [14]. Furthermore, there was a small but statistically significant difference in the indication for TE surgery, with slightly more patients undergoing surgery for infection-related problems in the control group at baseline. However, there were no differences in the baseline-to-follow-up comparisons in either group, suggesting that the indication for TE had no impact on the decrease in PTH rates in the intervention group (Table 2). While outpatient surgery was slightly more common among the control group centres at baseline, in- versus outpatient TE is not a factor that affects PTH [29].

Third, this project was not initially planned as an intervention study but as a QIP. This may have led to a less-controlled study environment. The study was not designed to determine which of the intervention activities had the greatest impact. However, a less-controlled environment can increase the generalizability of the findings to guide similar QIPs elsewhere.

Finally, our follow-up time was limited to 1 year. It would have been advantageous to have a longer follow-up to evaluate the sustainability of the decrease in PTH rates. This was not feasible because of the retrospective nature of the data, which were taken mainly from the NPR. We wanted to present only complete years to avoid possible seasonal differences in PTH rates. The sustainability of the decrease in PTH rates would be an interesting subject for future studies.

## Conclusions

The rates of postoperative haemorrhage, a major complication after tonsillectomy, can be reduced with a QIP. A national quality register can be used not only to identify areas for improvement, but also to evaluate the impact of an improvement project.
